# Using a Multi-Layer Stacked AlGaN/GaN Structure to Improve the Current Spreading Performance of Ultraviolet Light-Emitting Diodes

**DOI:** 10.3390/ma13020454

**Published:** 2020-01-17

**Authors:** Yanli Wang, Peixian Li, Xinyu Zhang, Shengrui Xu, Xiaowei Zhou, Jinxing Wu, Wenkai Yue, Yue Hao

**Affiliations:** 1 Wide Bandgap Semiconductor Technology Disciplines State Key Laboratory, School of Advanced Materials and Nanotechnology, Xidian University, Xi’an 710071, China; ylwang055065@163.com (Y.W.); xwzhou@mail.xidian.edu.cn (X.Z.); Jinxing_Wu_Xidian@163.com (J.W.); yuewenkai888@gmail.com (W.Y.); 2 Wide Bandgap Semiconductor Technology Disciplines State Key Laboratory, School of Microelectronics, Xidian University, Xi’an 710071, China

**Keywords:** ultraviolet light-emitting diodes (UVLEDs), current crowding, modulation-doped, APSYS, AlGaN/GaN

## Abstract

To obtain excellent current spreading performance of ultraviolet light-emitting diodes (UVLEDs), a 60-period stacked Si modulation-doped n-AlGaN/u-GaN structure is proposed to replace the traditional n-AlGaN structure. The high-resolution X-ray diffraction ω-scan rocking curves show that the periodic growth of AlGaN and GaN layers plays a positive role in reducing dislocation density. Compared with the conventional UV light-emitting diodes (LEDs), light emission micrographs of devices with a multi-layer stacked n-AlGaN/u-GaN structure reveal higher brightness and a more uniform distribution. In addition, the output power and external quantum efficiency under a 20-mA injection current are increased by 22% and 26.5%, respectively. Experimental and simulation results indicate that a multi-layer stacking structure can alleviate the current crowding effect in four ways: (1) a reduction in dislocation density; (2) replacement of quasi-two-dimensional electron transport with electronic bulk transport to enhance electron mobility; (3) an increase in electron concentration without improving the impurity concentration; and (4) a weakening of the electron scattering effect by reducing the impurity concentration.

## 1. Introduction

Ultraviolet light-emitting diodes (UV-LEDs) have received positive attention from scholars and companies around the world to due to their properties, such as: high switching speeds; lack of heat radiation; their non-toxic, uniform illumination; high energy; and long service life [1,2,3,4]. However, there are still many obstacles to overcome to further improve their photoelectric performance, one of which is the current crowding effect [5]. Since the positive and negative electrodes in the lateral UV-LEDs are located on the same side, the current distribution of the whole device is severely uneven [6]. On the one hand, the in-plane non-uniformity of luminous intensity and local overheating may appear in the quantum well regions [7,8], reducing the internal quantum efficiency and reliability of devices [6]. On the other hand, because the current crowding effect occurs near the electrodes, the optical loss caused by metal electrodes’ absorption will cut down the optical extraction efficiency [9]. In addition, the current crowding effect causes the forward voltage to rise, thus lowering the electro-optical conversion efficiency [10]. Consequently, promoting current spreading is one of the most important ways of enhancing the electrical and optical performance of UV-LEDs [11,12].

Over the past decade, various methods have been proposed to solve the current crowding problem. For instance, LEDs with a vertical conducting structure, as reported by Wang et al., have shown a much lower current crowding effect and better heat dissipation [13,14]. Kim et al. and Sheu et al. proved that the modified p-pad electrodes improve device reliability by facilitating uniform current flow [15,16]. Apart from these, the insertion of a current blocking layer beneath the p-pad electrode [17,18], the transparent conductive layer, and the current spreading layer is also able to promote uniform current spreading [19,20,21,22,23].

In addition to the abovementioned methods, the current spreading length equation shows that a low resistivity n-type layer can minimize the current crowding effect [4]. Wen et al. used several periodically modulation-doped 2.5-nm AlGaN/2.5-nm GaN superlattices in the p-GaN and n-GaN layers to reduce lateral resistivity [24]. However, the light-emitting efficiency of devices with the modulation-doped structure was 10% lower than that of the traditional devices. It is more difficult to control the growth of the superlattice because of the higher growth rate of the n-type layer. In this paper, we replace the conventional n-AlGaN layer with a 60-period stacked Si modulation-doped 20-nm n-AlGaN/15-nm u-GaN structure to obtain a well-distributed current. A multi-layer stacked n-AlGaN/u-GaN structure not only reduces the dislocation density, but also improves the electron concentration, weakens electron scattering, and increases electron mobility [25,26]. Here, it is worth noting that devices with a multi-layer stacking structure exhibit a 22% and 26.5% enhancement in the output power and external quantum efficiency (EQE), respectively, under a 20-mA injection current. Moreover, higher brightness and a more uniform light distribution appear in the optical emission micrograph of the multi-layer stacked structure.

## 2. Experiments

**Figure 1:**
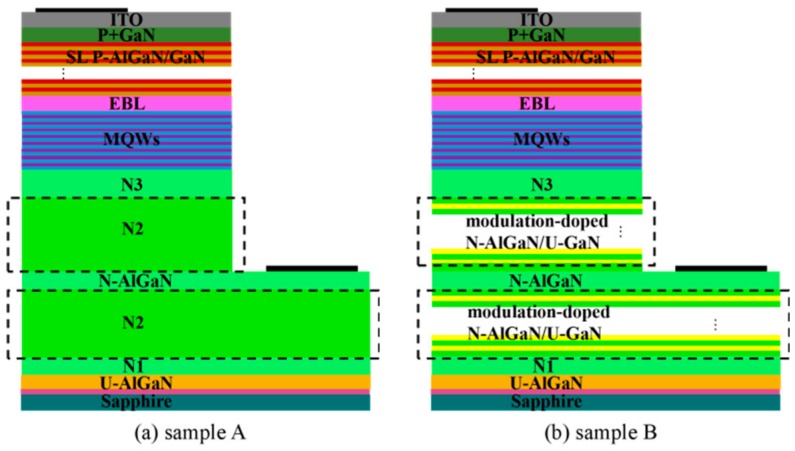
Schematic structures of the (**a**) conventional Sample A and (**b**) Sample B with a multi-layer stacking structure.

As shown in Figure 1, the epitaxial structure (sample A) was based on a c-plane (0001) 2-inch sapphire substrate, followed by a 25-nm magnetron sputtering AlN buffer layer, and then a 3 μm undoped Al_0.02_Ga_0.98_N layer. The n-type layers consisted of a 460-nm Si-doped N1-Al_0.02_Ga_0.98_N layer (n-doping = 5 × 10^18^ cm^−3^), a 1050-nm N2-Al_0.02_Ga_0.98_N (N+) layer (n-doping = 1 × 10^19^ cm^−3^), a 250-nm N-Al_0.02_Ga_0.98_N layer, a 1050-nm N2-Al_0.02_Ga_0.98_N (N+) layer, and a 460-nm N3-Al_0.02_Ga_0.98_N layer. The N-AlGaN layer was the contact layer and divided the N2-Al_0.02_Ga_0.98_N layer into two parts: the upper and lower part of the negative electrode. On top of the n-type layers, multiple quantum wells (MQWs) with eight 2-nm In_0.01_Ga_0.99_N wells embedded in nine 10-nm Al_0.08_Ga_0.92_N barriers, a 20-nm p-Al_0.2_Ga_0.8_N EBL (Electron barrier layer) (p-doping = 1 × 10^20^ cm^−3^), 30-period p-Al_0.16_Ga_0.84_N (3 nm)/u-GaN (2 nm) superlattices (p-doping = 1 × 10^20^ cm^−3^), and a 20 nm p+-GaN layer were grown. Sample B was identical to Sample A except for the n-type layers. In Sample B, the N2 layers above and below the cathode adopt a 30-period 20-nm n-Al_0.02_Ga_0.98_N/15-nm u-GaN modulation-doped structure.

After the epitaxial layers were grown, the wafers were partially etched by an inductively coupled plasma (ICP) process until the N-AlGaN contact layer and the Indium Tin Oxide (ITO) film were evaporated onto the top of the p+-GaN layer as the current spreading layer. Then, Gr/Ni/Au metal electrodes were deposited onto the N-AlGaN contact layer and the ITO film as the cathode and anode, respectively. The total thickness and diameter of the metal electrodes were 1.2 μm and 70 μm, respectively. Finally, the processed wafers were diced into bare die with a size of 325 μm × 300 μm.

## 3. Results and Discussion

### 3.1. HRXRD and Raman Images

**Figure 2:**
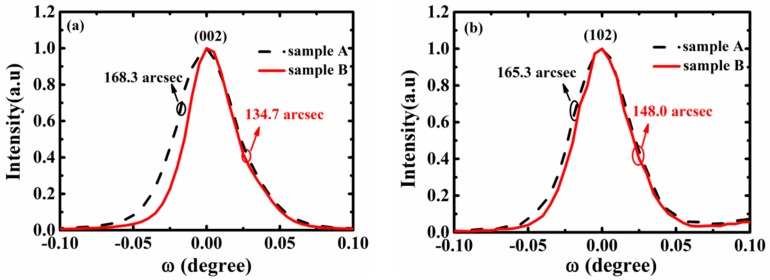
High-resolution XRD (HRXRD) ω-scans of the (**a**) (002) reflection and (**b**) (102) reflection for both samples.

Figure 2 shows the high-resolution X-ray diffraction (HRXRD) ω-scan rocking curves of the (002)/(102) reflection for both samples. The full width at half a maximum (FWHM) of the (002) reflection is 134.7 arcsec for sample B—narrower than sample A, whose value is 168.3 arcsec. The FWHMs of the (102) reflection for Samples A and B are 165.3 arcsec and 148.0 arcsec, respectively. The screw dislocation density was estimated to be 5.692 × 10^7^ cm^−2^ and 3.65 × 10^7^ cm^−2^ from the corresponding FWHM values [27], while the edge dislocation density was estimated to be 1.45 × 10^8^ cm^−2^ and 1.163 × 10^8^ cm^−2^ for Samples A and B, respectively. Screw dislocation is the preferred channel for a recombination current [28], and the tunneling current associated with dislocation can result in a leakage current [29], especially at a high injection current. Therefore, Sample A, with a larger dislocation density, produces a more serious current crowding effect than Sample B. Furthermore, dislocation will act as the non-radiative recombination center, reducing the internal quantum efficiency of devices [30].

**Figure 3:**
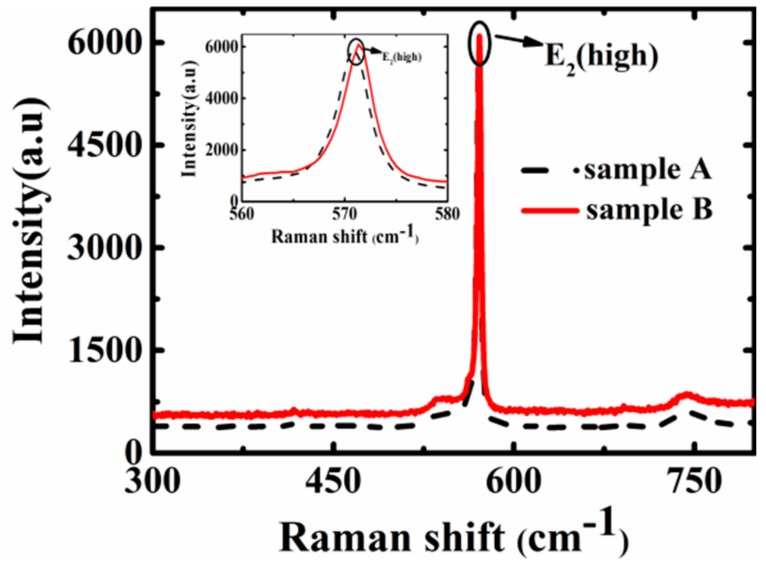
Raman spectra of both samples (the inset is a partially enlarged view of the spectra).

For the sake of studying the effect of a multi-layer stacked n-AlGaN/u-GaN structure on the stress of devices, the Raman spectra of both samples were taken by the confocal Jobin Yvon LabRam HR800 (HORIBA FRANCE SAS). As shown in Figure 3, the E_2_ (high) mode peaks of Samples A and B are 570.8 cm^−1^ and 571.3 cm^−1^, while the corresponding stresses are 0.86 GPa and 1 GPa, respectively. The compressive stress in Sample B is slightly higher than that in Sample A. It is well known that dislocation can release stress [31], so the stress state is consistent with the results of HRXRD.

### 3.2. Micrographs of Light Emission and APSYS Simulations

**Figure 4:**
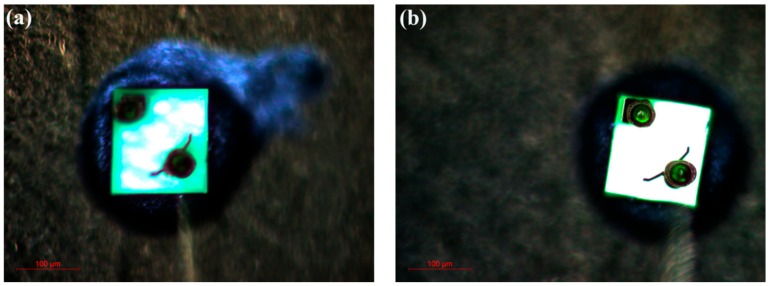
Micrographs of light emission for (**a**) Sample A and (**b**) Sample B.

Figure 4a,b are the optical emission micrographs for Samples A and B at a 1 mA injection current, respectively. As shown in Figure 4, there is a strong luminous intensity in the center area of Sample A, and a weak luminous intensity near the electrodes. However, compared with Sample A, higher brightness and more uniformly distributed light occur on the entirety of Sample B, which can mainly be attributed to the multi-layer stacked n-AlGaN/u-GaN structure. Due to the energy band discontinuity and polarization effect, channels are formed at the AlGaN/GaN heterojunction interface, where a large number of electrons will accumulate, thereby converting the electronic bulk transport into quasi-two-dimensional transport and promoting uniform current spreading [32,33]. Additionally, because ionized impurities in the materials have a scattering effect on carriers and thus reduce carrier mobility, the undoped GaN layer in the multi-layer stacking structure allows for higher electron mobility than the traditional n-AlGaN layer, which will enhance the current spreading capability of the devices.

**Figure 5:**
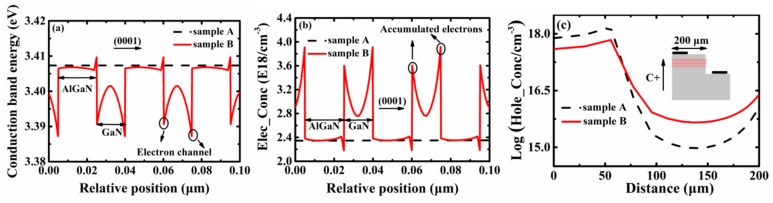
(**a**,**b**) are the calculated partial conduction band energy and electron concentration diagrams of both samples in the AlGaN/GaN structure; (**c**) is the hole concentration diagram along the x direction of both samples in the first quantum well.

To further explore the internal mechanisms of a multi-layer stacked n-AlGaN/u-GaN structure that promotes uniform current spreading, the partial conduction band energy and electron concentration in the AlGaN/GaN structure, and the hole concentration in the first quantum well of both samples, were calculated by the advanced physical model of semiconductor devices (APSYS) [34], which includes the tunneling and heterojunction models. The operating temperature, the Shockley–Read–Hall recombination lifetime, and the screening factor were set to 300 K, 100 ns, and 20%, respectively. As shown in Figure 5a, the energy band discontinuity of the AlGaN and GaN layers leads to the barrier and well structure in the multi-layer stacking structure, and charges caused by the polarization effect will pull down the conduction band at the heterojunction interface to form electron channels. Thus, a large number of electrons accumulate in the electron channels in Figure 5b, which will transform the electronic bulk transport into quasi-two-dimensional transport to improve electron mobility. The movement of the electron ionized by the n-AlGaN layers towards the u-GaN layers breaks the ionization equilibrium to promote donor ionization, thus improving the electron concentration without increasing the impurity concentration. Moreover, the undoped GaN layers have a weak scattering effect on accumulated electrons, which will further increase electron mobility. For the n-type semiconductors, the relationship between conductivity, electron concentration, and electron mobility is:

σ_n_ = neμ_n_, (1)

where σ_n_ is the electron conductivity, n is the electron concentration, and μ_n_ is the electron mobility. It can be seen from the above formula that increasing electron concentration and mobility can improve the conductivity of the devices. High conductivity will alleviate the current crowding effect of Sample B [4]. As shown in Figure 5c, the hole concentration of Sample B is lower than that of Sample A under the anode, but higher in other regions. Therefore, the light absorption of Sample B by the anode is relatively small, and the current density distribution in the active region is more uniform, which will make the luminous intensity well-distributed.

### 3.3. Photoelectric Characteristics 

**Figure 6:**
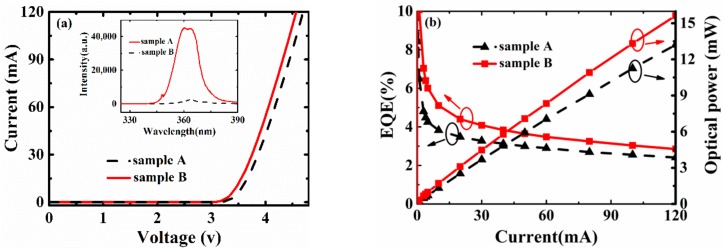
(**a**) The measured I–V characteristics of both samples (the inset shows the PL (Photoluminescence) spectrum at room temperature); (**b**) the optical output power and external quantum efficiency of both samples.

The I–V characteristics of both samples are shown in Figure 6a. The experimental results show that the forward voltage (Vf) of Samples A and B at a 20-mA injection current are 3.74 V and 3.52 V, respectively. The decrease in Vf of Sample B is due to its increased conductivity, which improves the electro-optical conversion efficiency. In the inset, the PL (Photoluminescence) spectrum of both samples was measured by a 325-nm laser diode at room temperature. The photoluminescence intensity of Sample B was much higher than that of Sample A. Figure 6b shows the results of the light’s output power as well as EQE. As shown in the figure, the output power of Samples A and B at 20 mA is 2.52 mW and 3.1 mW, respectively. A 22% increase in the output power of Sample B suggests that the well-distributed current and low dislocation density are beneficial to improving the luminous efficiency. Also, because a strong current spreading can alleviate the thermal effect under long-term operation [35], the output power of Sample B is larger than that of Sample A as the injection current increases. Other than this, compared with Sample A, there is a 26.5% enhancement in the EQE of Sample B, proving that high power UV-LEDs are obtained.

## 4. Conclusions

In this study, two different devices were grown by Metal Organic Chemical Vapor Deposition. One was the traditional UV-LEDs, and the other was the UV-LEDs with a 60-period stacked Si modulation-doped n-AlGaN/u-GaN structure. Compared with the traditional UV-LEDs, the latter improves the current spreading performance of the devices in four ways. Firstly, the lower dislocation density corresponds to a weaker current crowding effect; secondly, the quasi two-dimensional electron transport enhances electron mobility; thirdly, the electron concentration is added to improve the conductivity; and fourthly, the reduction of the impurity concentration lessens the electron scattering effect. The light emission micrographs of devices with a multi-layer stacked n-AlGaN/u-GaN structure revealed higher brightness and a more uniform distribution. Our experimental results show that higher-performance devices with a 22% and 26.5% enhancement in the output power and EQE, respectively, were obtained.
